# Layered Cathode with Ultralow Strain Empowers Rapid‐Charging and Slow‐Discharging Capability in Sodium Ion Battery

**DOI:** 10.1002/advs.202404701

**Published:** 2024-06-28

**Authors:** Maolin Yang, Ziwei Chen, Zhongyuan Huang, Rui Wang, Wenhai Ji, Dong Zhou, Tao Zeng, Yongsheng Li, Jun Wang, Liguang Wang, Tingting Yang, Yinguo Xiao

**Affiliations:** ^1^ School of Advanced Materials Peking University Shenzhen Graduate School Shenzhen 518055 P. R. China; ^2^ Department of Engineering University of Cambridge Cambridge CB30FS UK; ^3^ Spallation Neutron Source Science Center Dongguan 523803 P. R. China; ^4^ School of Advanced Energy Shenzhen Campus of Sun Yat‐sen University Shenzhen 518107 P. R. China; ^5^ School of Innovation and Entrepreneurship Southern University of Science and Technology Shenzhen 518055 P. R. China; ^6^ College of Chemical and Biological Engineering Zhejiang University Hangzhou 310000 P. R. China; ^7^ Ernst Ruska‐Centre for Microscopy and Spectroscopy with Electrons Forschungszentrum Jülich GmbH 52428 Jülich Germany

**Keywords:** layered cathode, rapid‐charging and slow‐discharging capability, ultralow strain

## Abstract

The development of the electric vehicle industry has spurred demand for secondary batteries capable of rapid‐charging and slow‐discharging. Among them, sodium‐ion batteries (SIBs) with layered oxide as the cathode exhibit competitive advantages due to their comprehensive electrochemical performance. However, to meet the requirements of rapid‐charging and slow‐discharging scenarios, it is necessary to further enhance the rate performance of the cathode material to achieve symmetrical capacity at different rates. Simultaneously, minimizing lattice strain during asymmetric electrochemical processes is also significant in alleviating strain accumulation. In this study, the ordered distribution of transition metal layers and the diffusion pathway of sodium ions are optimized through targeted K‐doping of sodium layers, leading to a reduction of the diffusion barrier and endowment of prominent rate performance. At a 20C rate, the capacity of the cathode can reach 94% of that at a 0.1C rate. Additionally, the rivet effect of the sodium layers resulted in a global volume strain of only 0.03% for the modified cathode during charging at a 10C rate and discharging at a 1C rate. In summary, high‐performance SIBs, with promising prospects for rapid‐charging and slow‐discharging capability, are obtained through the regulation of sodium layers, opening up new avenues for commercial applications.

## Graphical abstract

This study designs a K^+^ anchored P2‐type sodium‐ion cathode Na_0.62_K_0.05_Ni_0.33_Mn_0.67_O_2_. The introduction of K^+^ disrupts the ordered arrangement of transition metal layers and optimizes the Na^+^ diffusion pathways, significantly enhancing its rate performance. Consequently, symmetric capacity delivery and ultralow structural strain are achieved under various symmetric and asymmetric charge–discharge rates, endowing the cathode with reversible rapid‐charging and slow‐discharging capability.

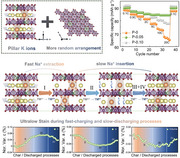

## 1 Introduction

In recent years, the rapid development of electric vehicles has made electric transportation increasingly mainstream.^[^ ^1^ ^]^ Among them, lithium‐ion batteries (LIBs) have always occupied a dominant position in the energy storage modules of electric vehicles due to their higher energy density.^[^ ^2^ ^]^ However, a significant drawback of electric vehicles compared to traditional internal combustion engine vehicles is the low charging speed, leading to range anxiety.^[^ ^3^ ^]^ A preferable solution to this problem is to introduce secondary batteries with rapid‐charging capability on the basis of existing LIBs, and require these batteries to release energy slowly during the discharging process in order to meet actual demands, i.e., possessing rapid‐charging and slow‐discharging capability.^[^ ^4^ ^]^

Specifically, the rapid‐charging and slow‐discharging capability requires excellent rate performance of both cathode and anode materials. This implies that under conditions where there is a large difference between the charge and discharge rates, both cathode and anode materials should exhibit more symmetrical capacities. On the other hand, it also requires lower structural strain throughout the entire asymmetric electrochemical process to mitigate detrimental stress accumulation.^[^ ^5^ ^]^ Among the candidate solutions, sodium‐ion batteries (SIBs) have received widespread attention due to their similar energy storage mechanism to LIBs.^[^ ^6^ ^]^ This similarity also implies that the rapid‐charging and slow‐discharging capability of SIBs is similarly limited by the cathode materials, although they already showed obviously enhanced rated performance compared to LIBs.^[^ ^7^ ^]^ Therefore, considering the energy density, cycle life, rate performance, and other performance indicators of cathode materials, layered oxides have emerged as one of the most competitive candidates due to their superior comprehensive advantages.^[^ ^8^ ^]^ Specifically, the focus of this research is P2‐type layered oxides with smoother sodium‐ion diffusion channels.^[^ ^9^ ^]^

Recently, researchers have made efforts to improve the ionic and electronic conductivity of P2‐type layered oxides through various approaches to achieve high‐rated cathodes, including both crystal structure modification (such as inert ion doping,^[^ ^10^ ^]^ superstructure regulation,^[^ ^11^ ^]^ and phase structure optimization^[^ ^12^ ^]^) and interface structure optimization (functional coating,^[^ ^13^ ^]^ regulation of advantageous crystal surface exposure,^[^ ^13^, ^14^ ^]^ and surface defect regulation^[^ ^15^ ^]^). Although these studies have significantly improved the rate performance of basic P2‐type layered oxides, they still struggle to meet the requirements of high‐rate capacity retention during rapid‐charging and slow‐discharging processes. Additionally, researchers have developed a series of layered cathode materials with almost zero strain.^[^ ^10^, ^16^ ^]^ However, the structural strain of these zero‐strain cathodes during the entire charge/discharge process often exceeds 1%. This level of strain still makes it difficult to adapt to the structural instability caused by the asymmetric electrochemical process during rapid‐charging and slow‐discharging. Therefore, it is a pressing matter of the moment to explore more suitable modification strategies. Compared to modification of the transition metal (TM) layers, modification of the sodium layers can directly influence the migration process of sodium ions in cathode materials, making it more likely to optimize the diffusion pathway of sodium ions.^[^ ^16^, ^17^ ^]^ Furthermore, selecting electrochemically inert ions is more beneficial for the stability of their structures, and layer optimization with pillar effects is expected to achieve ultralow strain cathode materials.

In this study, potassium ions with pillar effects and electrochemical inertness are selected as doping elements in the sodium layers of layered cathode, and Na_0.62_K_0.05_Ni_0.33_Mn_0.67_O_2_ (denoted as P‐0.05) and Na_0.57_K_0.10_Ni_0.33_Mn_0.67_O_2_ (denoted as P‐0.10) cathode materials are successfully synthesized on the basis of P2‐type Na_0.67_Ni_0.33_Mn_0.67_O_2_ (denoted as P‐0) substrates using a simple solid‐phase synthesis method. Through powder neutron diffraction (NPD), aberration‐corrected scanning transmission electron microscopy (STEM), and X‐ray absorption spectroscopy studies, it was found that the incorporation of potassium ions reduced the proportion of unfavorable Ni@Mn_6_ octahedral superstructures in the nickel–manganese‐based cathode, thereby reducing the energy barrier for sodium ion diffusion. Further investigation with in/ex situ X‐ray diffraction (XRD) techniques and electrochemical characterization indicated that P‐0.05 had a more optimal sodium ion diffusion pathway and lower structural strain. GITT tests showed that the sodium ion diffusion coefficient of P‐0.05 is an order of magnitude higher than that of P‐0 counterpart. Benefiting from these structural optimizations, P‐0.05 exhibited extremely excellent rate performance, providing 94% of the capacity at a rate of 0.1C under a 20C rate. Most importantly, at a charge rate of 10C and discharge rate of 1C, the overall volume strain of P‐0.05 was only 0.03%, achieving an ultralow‐strain cathode. This study offers novel insights into achieving the rapid‐charging and slow‐discharging capability of SIBs cathode materials.

## 2 Results

### 2.1 Pristine Structure Characterization

Utilizing a straightforward solid‐state reaction method, this research adeptly synthesized classical cathode of Na_0.67_Ni_0.33_Mn_0.67_O_2_ (denoted as P‐0) as well as gradient K‐doping cathodes of Na_0.62_K_0.05_Ni_0.33_Mn_0.67_O_2_ (denoted as P‐0.05), and Na_0.57_K_0.10_Ni_0.33_Mn_0.67_O_2_ (denoted as P‐0.10). As shown in Figure S1 (Supporting Information), all synthesized samples exhibit a layered SIBs cathode with a characteristic micrometer‐sized plate‐like morphology. The atomic ratios obtained from the energy dispersive X‐ray spectroscopy (EDS) elemental mapping results of each sample are generally consistent with the designed ratios (Figure S2, Supporting Information). Traditional Ni–Mn‐based layered cathode materials for SIBs exhibit complex superstructures, including ordered arrangements of Na‐vacancies and TMs, posing challenges for conventional XRD techniques in accurately delineating their crystalline architecture. Therefore, despite all samples displaying typical P2 phase diffraction peaks as shown in Figure S3 (Supporting Information), the variations in relative diffraction peak intensities and the appearance of Na‐vacancy ordered superstructure peaks indicate the necessity for additional techniques to achieve a more accurate analysis of their crystal structure. The distinct difference in the neutron scattering cross‐sections between Ni and Mn elements enabled neutron diffraction to emerge as a powerful tool for structural elucidation of the cathode materials engineered in this investigation.

**Figure 1:**
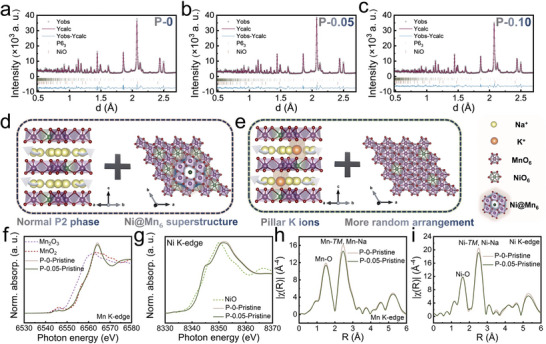
a–c) The NPD and corresponding Rietveld refinement results of the a) P‐0, b) P‐0.05, and c) P‐0.10. d, e) Crystal structure schematics of d) P‐0, e) P‐0.05, and P‐0.10 along the [110] and [001] zone axes. f, g) Normalized XANES spectra at the f) Mn *K*‐edge and the g) Ni *K*‐edge at the pristine state of P‐0 and P‐0.05. h, i) The k^3^ weighted EXAFS spectra at the h) Mn *K*‐edge and the i) Ni *K*‐edge after Fourier transformation at the pristine state of P‐0 and P‐0.05.

**Figure** **1**a–c showcase the NPDF patterns for the samples P‐0, P‐0.05, and P‐0.10. Employing Rietveld refinement, intricate structural details such as cell parameters and atomic positions were meticulously derived for these samples, with the aggregated data presented in Tables S1–S3 (Supporting Information). The sharpness of the diffraction peaks and the superior fit quality, as evidenced in Figure 1a–c, affirm the high fidelity of the refinement outcomes. The layered cathode lattice structures across all samples were precisely described using a *P6_3_* symmetry crystal cell. The NPD patterns indicated the exclusive presence of minor NiO impurities, aside from which no other impurities were detected, validating the efficacious doping of K^+^. The integration of Rietveld refinement outcomes for P‐0.05 with EELS data (Figure S4, Supporting Information) definitively located the K^+^ doping predominantly at the Nae sites.

Remarkably, the introduction of K^+^ induced negligible alterations to the lattice's *c*‐axis, likely attributed to K^+^ carrying a single positive charge and occupying the expansive spaces within the Na layers. As depicted in Figure 1d, the projection of the P‐0 along the [110] direction paralleled that of conventional P2‐type layered cathodes, yet unveiled a distinctive Ni@Mn_6_ hexamer configuration in the [001] direction, a superstructure critical to the modulation of sodium ion diffusion kinetics. Notably, an increase in K^+^ doping resulted in a significant reduction in the Ni@Mn_6_ proportion. In the *P6_3_* unit cell, there are three types of sites for transition metals: *2b1*, *2b2*, and *2a*. The *2b1* site is the central position of the Ni@Mn_6_ hexagonal rings, while the other two are peripheral sites. When all Ni atoms occupy the *2b1* sites, the sample is considered fully ordered. Thus, the ratio *OCC*. Ni*_2b1_* / (*OCC*. Ni*_2b1_* + *OCC*. Ni*_2b2_* + *OCC*. Ni*_2a_*) can be used as a metric for the degree of order in the transition metal layer. The closer this ratio is to 100%, the more ordered the transition metal layer is; the closer it is to 33%, the more disordered the layer is. As detailed in Tables S1–S2 (Supporting Information), the Ni@Mn_6_ content in the P‐0.05 decreased by 12.6% compared to P‐0. However, a further escalation in K^+^ doping conversely led to an increase in this ratio, likely due to an augmented presence of NiO impurities.

Figure 1e illustrates that the strategic localization of K^+^ within the sodium layers not only conferred structural stability to the layered matrix but also subtly modulated the sodium ion diffusion pathways and indirectly influenced the superstructural arrangement of the transition metal layers. This nuanced interplay significantly impacted the sodium ion diffusion kinetics, ultimately enhancing the electrochemical performance of the cathode materials.

Furthermore, X‐ray absorption spectroscopy (XAS) is performed to reveal the local coordination environment of the initial samples. Figure 1f–g depict the normalized X‐ray absorption near‐edge structure (XANES) spectra for P‐0 and P‐0.05, specifically focusing on the Mn and Ni *K*‐edges, respectively. The primary peak (white line) in both the Mn and Ni *K*‐edges arises from the electron transition originating from the *1s* to *4p* orbitals. By analyzing the edge and white line features of P‐0 and P‐0.05 in comparison with those of standard reference oxides of Mn, it is evident that K^+^ doping does not alter the oxidation state of Mn within these materials; both consistently exhibit a +4 valence. The resemblance in the pre‐edge peaks ≈ 6545 eV for both materials underscores a similar octahedral oxygen coordination environment, attributed to the quadrupole‐allowed yet dipole‐forbidden electronic transitions from *1s* to *3d* orbitals. Likewise, the data presented in Figure 1g confirm that Ni maintains an identical valence state of ≈ +2 in both P‐0 and P‐0.05. Importantly, the comparison reveals that the amplitude of the white line at the *K*‐edge of both Mn and Ni is more pronounced in P‐0 than in P‐0.05. This distinction indicates a variance in the elemental distribution surrounding the central TM sites between these two samples, signifying a difference in the degree of order within the Ni@Mn_6_ superstructure present in their TM layers. To elucidate the local structural differences of Mn and Ni between the samples, their extended X‐ray absorption fine structure (EXAFS) spectra were subjected to k^3^‐weighted Fourier transformation. Figure 1h–i display the Fourier‐transformed EXAFS spectra of P‐0 and P‐0.05 at the Mn and Ni *K*‐edges in their pristine state. Notably, the second peak in the k^3^‐weighted EXAFS spectra at both the Mn and Ni *K*‐edges is less pronounced for P‐0.05 than for P‐0, suggesting a more homogeneous distribution of Ni and Mn within the TM layers of P‐0.05. This observation indicates that P‐0 exhibits a higher degree of order in the Ni@Mn_6_ superstructure, corroborating the findings from NPD refinement.

**Figure 2:**
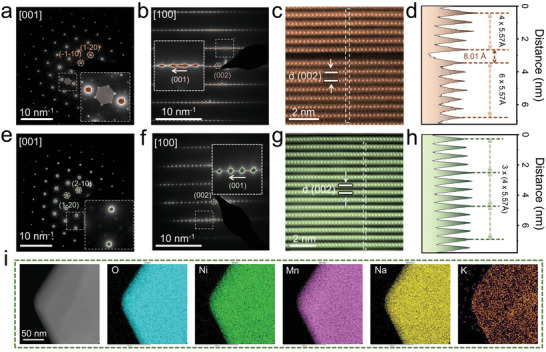
a, b) SAED patterns of P‐0 observed along a) [001] and b) [100] zone axes (Insets are the enlarged images of the corresponding area, respectively). c)HAADF‐STEM image of P‐0 along the [100] zone axis. d) Line scan profile along the corresponding rectangle in (c). e, f) SAED patterns of P‐0.05 were observed along e) [001] f) [100] zone axes (Insets are the enlarged images of the corresponding area, respectively). g) HAADF‐STEM image of P‐0.05 along the [100] zone axis. h) Line scan profile along the corresponding rectangle in (g). i) EDS elemental mapping of P‐0.05 at the pristine state.

The microscopic structures of the material were thoroughly investigated using transmission electron microscopy (TEM) and aberration‐corrected STEM. In **Figure** **2**a, the selected area electron diffraction (SAED) along the [001] zone axis of P‐0 reveals distinct hexagonal crystal patterns and numerous small diffraction spots, believed to be associated with the Ni@Mn_6_ hexagonal superstructure within the *ab* plane.^[^ ^18^ ^]^ Due to the higher content of Ni@Mn_6_ hexagonal superstructure in P‐0, the diffraction spots of the new period are more prominent. Additionally, the SAED along the [100] zone axis for P‐0 exhibits elongated patterns along the [001] direction, as shown in Figure 2b. This irregular pattern suggests varying interlayer spacings along the *c*‐axis for P‐0 (Figure 2c), and this nonuniformity is often pervasive throughout the material (Figure S5a, Supporting Information). For layered SIB cathodes, such nonuniform interlayer spacing leads to stress accumulation between layers, compromising structural stability during electrochemical cycling.

Furthermore, the line scanning profile (Figure 2d) along the rectangle within the high‐angle annular dark‐field STEM (HAADF‐STEM) image reveals noticeable non‐uniformities in interlayer spacing. However, with K^+^ intercalation in P‐0.05, the microstructure is profoundly influenced. On one hand, K^+^ doping affects the arrangement of the TM layers. Specifically, in the SAED along the [001] zone axis, almost no visible diffraction pattern of the new period is observed in P‐0.05, indicating a lower content of the Ni@Mn_6_ hexagonal superstructure. Figure S6a,b (Supporting Information) shows the simulated electron diffraction pattern along the [001] zone axis based on the crystal structure obtained from the Rietveld NPD refinement. The simulated results are in good agreement with the experimental observations (Figure **2**a,e). On the other hand, due to potassium ions anchoring in the sodium layers, P‐0.05 exhibits a significantly uniform layered structure (Figures 2e–g; Figure S5b, Supporting Information). As demonstrated in Figure 2h, the interatomic distance mapping for P‐0.05 is predominantly located at d = 5.57Å. The exceptional nature of this structure will profoundly impact subsequent electrochemical processes. Additionally, energy‐dispersive X‐ray spectroscopy (EDS) spectra of P‐0.05 particles further confirm the uniform potassium ion doping throughout the material particles, contributing to the formation of a uniform interlayer spacing.

### 2.2 Electrochemical Performance

The incorporation of potassium ions into the layered cathode structure significantly influences its electrochemical characteristics. In an effort to delve deeper into the benefits of this modification, the synthesized samples were paired with a metallic sodium anode to assemble a half‐cell, which was then subjected to an array of electrochemical evaluations. The voltage window for all tests was set in the range of 2–4.1 V.

**Figure 3:**
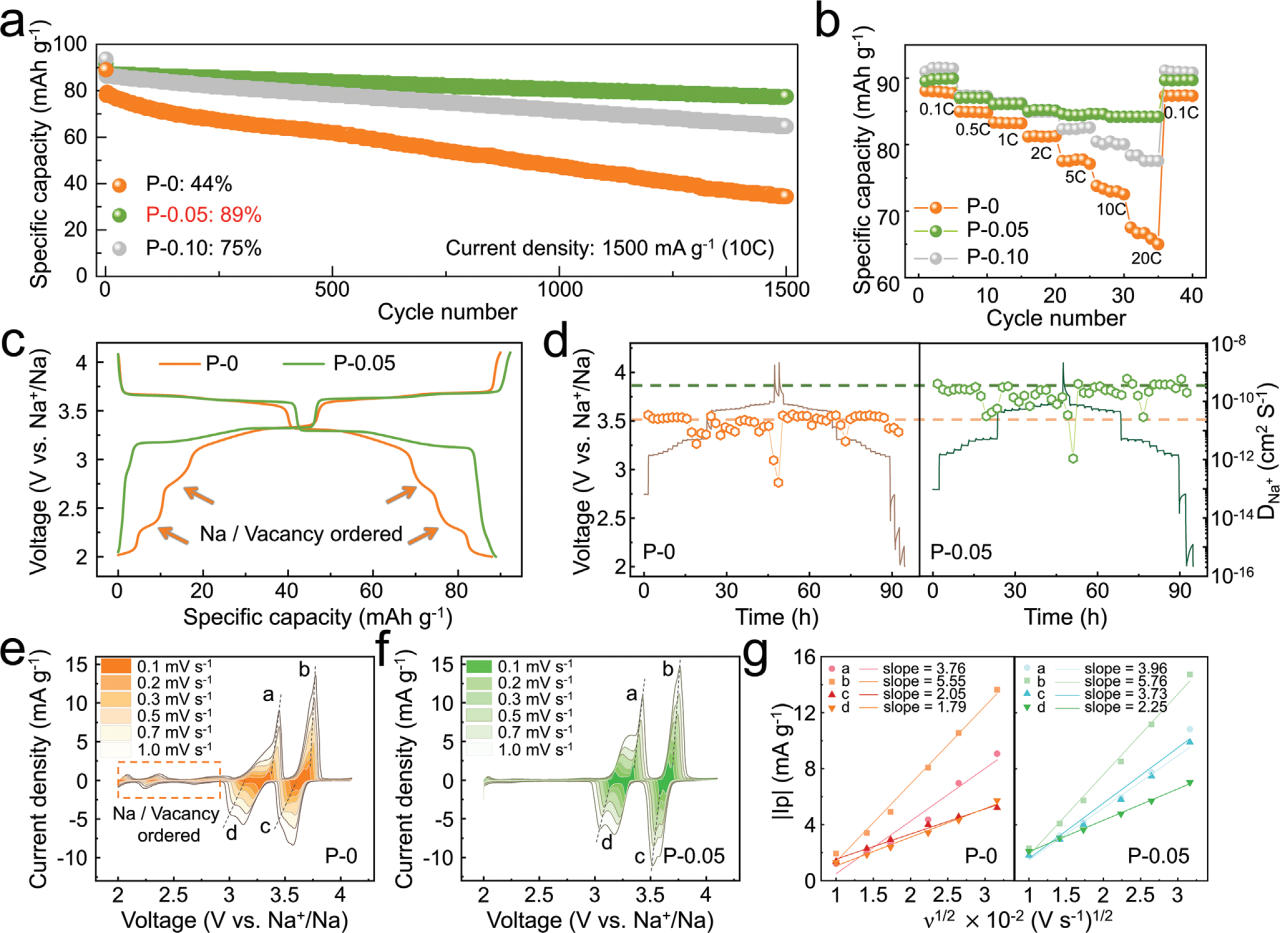
Electrochemical performance. a) Long‐term cycling retention of P‐0, P‐0.05, and P‐0.10 at rates of 10C. b) The rate performance comparison of P‐0, P‐0.05, and P‐0.10 at different rates. c) Typical GCD curves of P‐0 and P‐0.05 within the voltage range of 2.0–4.1 V, showcasing the second cycle at a rate of 0.1C. d) Charged/discharged GITT curves and corresponding calculated D_Na+_ of P‐0 and P‐0.05. e, f) Variable scan rate CV curves from 0.1 to 1 mV s^−1^ of e) P‐0 and f) P‐0.05. g) The results of linear fitting for the absolute value of peak current intensity (|Ip|) plotted against the square root of the scan rate (ν^1/2^) for P‐0 and P‐0.05.

Initially, longevity tests at a 1C rate (Figure. S7, Supporting Information) reveal that after 100 cycles of galvanostatic charge–discharge (GCD), all materials demonstrated commendable cycling stability. This outcome suggests that the TM layered framework can structurally adapt over time during the gradual incorporation and release of sodium ions, effectively mitigating the detriments of structural evolutions. Notably, one of the key advantages of SIBs over their lithium‐ion counterparts is their superior rate capability. With this in mind, this work further explored the cycling stability of all the samples under the demanding conditions of a 10C rate. These tests (illustrated in **Figure** **3**a) showed that doping with potassium ions substantially improves the material's performance during high‐rate cycling. Remarkably, the P‐0.05 maintained an 89% capacity retention even after 1500 cycles at a 10C rate, vastly surpassing the 75% retention of the P‐0.10 and the 44% retention of the undoped P‐0.

Furthermore, rate capability assessments (shown in Figure 2c) validated the exceptional performance of P‐0.05 under high‐rate conditions, with the material retaining 94% (compared to its performance at a 0.1C rate) of its capacity at a 20C rate. This highlights P‐0.05′s potential for applications requiring rapid charging, offering an enticing combination of rapid charge and slow discharge capabilities.

To gain a deeper understanding of the outstanding rate performance of P‐0.05, we conducted GCD tests on the P‐0 and P‐0.05 at a 0.1C rate (illustrated in Figure 3d–e), alongside d*Q*/d*V* curve analysis (Figure S8, Supporting Information). Both samples exhibited redox peaks above 3 V, though the P‐0 also presented minor plateaus between 2–2.5 V, indicating the rearrangement of a Na‐vacancy‐ordered structure within the sodium layer. As illustrated in Figure S9a (Supporting Information), extensive literature reports demonstrate that Na_δ_[Ni_1/3_Mn_2/3_]O_2_ exhibits distinct sodium layer ordering patterns at various sodium concentrations. For instance, at δ = 2/3, this specific arrangement, referred to as “large zigzag” (LZZ), is identifiable in XRD patterns by two minor peaks located at 27°–28°.^[^ ^19^ ^]^ Ex situ XRD patterns in Figure S9b (Supporting Information) show that the initial states of both samples display the LZZ configuration in Na‐layer. Upon charging to 4.1 V, these samples undergo a structural transition to a row‐type ordering, where sodium ions align in rows on either the Na_e_ or Na_f_ sites within a single layer, resulting in the disappearance of the characteristic diffraction peaks at 27°–28° (Figure S9c, Supporting Information). During discharging, the reintroduction of Na ions leads to the recovery of the LZZ ordering in the Na‐layer within P‐0 (Figure S9d, Supporting Information). Conversely, the disappearance of the ordered structural peaks in P‐0.05 indicates a lack of re‐establishment of the LZZ order, a phenomenon that aligns with the absence of discharge plateaus associated with the reordering process in its GCD curves (Figure 3c). This suggests that the rivet of K^+^ at the Na_e_ sites within the Na‐layers modifies the diffusion pathways during the insertion of sodium ions. Importantly, this ordered arrangement of the Na‐layers signifies slower sodium ion diffusion dynamics within the layer.

Kinetic studies through the Galvanostatic Intermittent Titration Technique (GITT) and calculations of the sodium ion diffusion coefficient D_Na_+ (with details provided in the **Experimental section**) revealed that D_Na_+ for P‐0.05 is higher than that of P‐0 by an order of magnitude (Figure 3d), suggesting markedly faster sodium ion diffusion kinetics for P‐0.05 throughout the charging and discharging processes. Additionally, variable scan rate Cyclic Voltammetry (CV) analysis, a method used to evaluate the electrochemical diffusion rates of cathode materials, showcased two pairs of redox peaks (peaks a/d and b/c) associated with nickel ion charge transfer for both samples. These absolute values of peak current intensity |Ip| demonstrated a linear relationship with the square root of the scan rate (ν^1/2^) (Figure 3e–f), indicative of diffusion‐controlled processes. According to the Randles–Sevcik equation, the slope of this linear relationship positively correlates with the sodium ion diffusion rate, further evidenced by the significantly steeper slopes for P‐0.05 compared to P‐0 (Figure 3g).^[^ ^20^ ^]^ This corroborates with GITT results, affirming P‐0.05′s enhanced sodium ion diffusion kinetics.

Collectively, these findings underscore the significant role of potassium ion doping in elevating the electrochemical performance of the material, particularly under high‐rate cycling conditions. P‐0.05 emerges as a superior candidate, showcasing exceptional capabilities vital for rapid‐charging applications in the realm of commercial batteries.

### 2.3 Charge Compensation Mechanism

**Figure 4:**
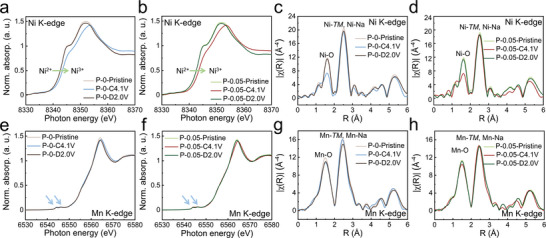
a, b) Normalized Ni *K*‐edge XANES at various charged/discharged states of a) P‐0 and b) P‐0.05. c, d) The k^3^ weighted Ni *K*‐edge EXAFS spectra after Fourier transformation at various charged/discharged states of c) P‐0 and d) P‐0.05. e, f) Normalized Mn *K*‐edge XANES at various charged/discharged states of e) P‐0 and f) P‐0.05. g, h) The k^3^ weighted Mn *K*‐edge EXAFS spectra after Fourier transformation at various charged/discharged states of g) P‐0 and h) P‐0.05.

To investigate the potential impact of structural optimization on the charge compensation mechanism of cathode materials, ex situ XAS tests were conducted on P‐0 and P‐0.05 at different charge and discharge states. As depicted in **Figure** **4**a–b, the white lines at the Ni *K*‐edge for both P‐0 and P‐0.05 exhibit shifts toward higher energy positions upon charging to 4.1 V and return to lower energy positions during discharge to 2.0 V. This behavior signifies the oxidation and reduction of Ni ions. Although both materials show a similar trend in Ni valence changes, distinct differences are observed in the amplitude changes of their white lines. Detailed analysis of the Fourier‐transformed EXAFS spectra in the 1.0–2.0 Å range at the Ni *K*‐edge (Figure 4c–d) reveals evident reduction and increase in amplitude. This points to changes in the first coordination shell around Ni, occupied by six O atoms. However, in the 2.0–3.0 Å range, the amplitude is more influenced by Na occupation due to the stable distribution of the TM layer. Notably, P‐0.05 keeps a stable response between the states of charging to 4.1 V and discharging to 2.0 V, possibly linked to the ordered rearrangement of Na^+^ and vacancy, consistent with the absence of a small plateau at 2–2.5 V observed in the GCD curves (Figure 3c).

Concerning Mn *K*‐edge XANES, no substantial shift is noted in the white lines for P‐0 and P‐0.05 during the charge–discharge process, yet their amplitudes undergo changes (Figure 4e–f). Fourier‐transformed EXAFS spectra analysis (Figure 4g–h) suggests that P‐0.05 exhibits better reversibility in the Mn‐O peak after the charge–discharge process compared to P‐0. This implies enhanced reversibility in the structural evolution during charge–discharge for P‐0.05. Furthermore, the evolution of the peak strength related to the second coordination shell around Mn is more moderate in P‐0.05, providing additional evidence for the improved reversibility of the electrochemical process.

## 3 Discussion

### 3.1 In Situ Structure Evolution at Low Rate

**Figure 5:**
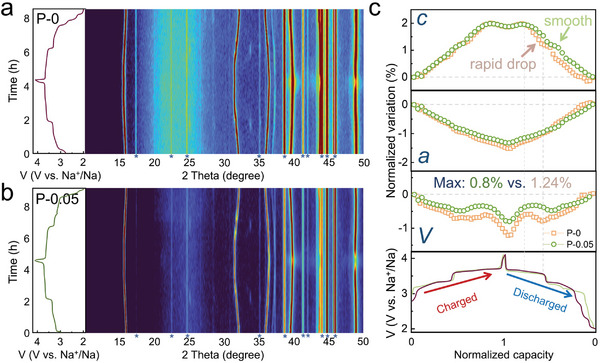
a, b) Contour plots of in situ XRD characterization and corresponding GCD curves for a) P‐0 and b) P‐0.05 during the first charged–discharged processes at the rate of 0.1C. Asterisks denote diffraction peaks of inactive cell components such as Be and Al etc., which do not interfere with the evolution of the cathode material during the electrochemical process. c) The corresponding evolution of refined parameters (lattice volume, lattice parameters *a* and *c*) during the first charged–discharged processes at the rate of 0.1C for P‐0 and P‐0.05.

To reveal the structural evolution mechanism of the cathode materials during charge and discharge processes, in situ XRD characterizations were conducted on P‐0 and P‐0.05 at 0.1C rate. As depicted in **Figure** **5**a–b, both samples exhibited typical solid‐solution reactions throughout the entire charge and discharge cycles, without observable generation of new phases. This type of solid‐solution reaction is generally considered relatively reversible. Further refinement using the Rietveld method was performed on the full spectra of both samples, and the evolution ratios of volume and lattice parameters *a* and *c* during charge and discharge processes relative to the initial state were extracted (Figure 5c).

During the charging process, as Na^+^ gradually extracts, the shielding effect for O–O repulsion between adjacent TM layers decreases, resulting in an expansion of the interlayer distance corresponding to an increase in the *c*‐axis. As Na^+^ further extracts from the Na‐layer, a typical P2‐O2 phase transition occurs at ≈4.2 V in Ni–Mn‐based materials, resulting in substantial lattice contraction, hence causing the *c‐*axis to contract over 4.0 V.^[^ ^21^ ^]^ Additionally, the anionic oxidation reactions in the high‐voltage region reduce the O–O repulsion between adjacent TM layers, leading to a slowdown in *c*‐axis expansion or even contraction.^[^ ^22^ ^]^ Simultaneously, due to the oxidation of Ni ions to a high‐valence state with a smaller radius, the contracted TMO_6_ octahedra causes a reduction in the *a*‐axis. The evolution trend during discharge is opposite to that during charging. Importantly, the evolution process of P‐0.05 appears to be more reversible. Notably, the GITT results suggest that the plateau phase observed after the discharge initiation indicates a favorable environment for sodium ion diffusion kinetics (Figure 3d). The massive sodiation within Na‐layers during this period caused significant changes in the P‐0 lattice structure, particularly along the *c*‐axis. However, in the case of P‐0.05, the presence of potassium ions within the sodium layers acts as an anchor, leading to a more stable and reversible evolution of the structure (the top part of Figure 5c). Additionally, benefiting from the anchoring effect of K^+^, P‐0.05 exhibits a minimal volume strain of only 0.8% throughout the entire charge and discharge process (in contrast, P‐0 experiences a volume strain of 1.24%), approaching nearly zero strain. Due to the ultralow volume strain of P‐0.05 during electrochemical cycling, it maintains an intact layered particle morphology after cycling (500 cycles at 10C), in contrast to P‐0, which exhibits typical interlayer cracks (Figure S10a–f, Supporting Information). Notably, P‐0.05 particle still exhibits a relatively uniform elemental distribution after 500 cycles at 10C, especially for K^+^ (Figure S11, Supporting Information). This indicates that K^+^ remains stably anchored within the structure during cycling, contributing to the ultralow structural strain. This extremely low structural strain is a crucial factor driving its outstanding performance in achieving rapid charging and slow discharging, a pivotal performance indicator in SIBs.

### 3.2 In situ Structure Evolutions at Various Rates

**Figure 6:**
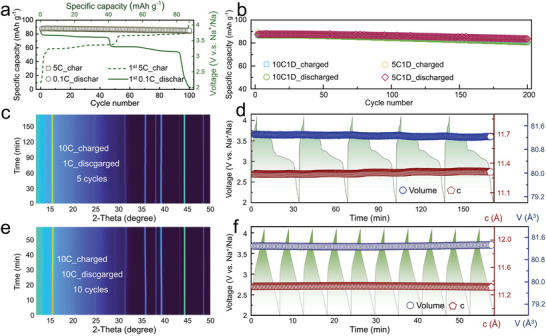
a–c) Long‐term cycling retention and first charged/discharged GCD curve of P‐0.05 at a charge rate of 5C and discharge rate of a) 0.1C, b) charge rate of 5C and discharge rate of 1C and charge rate of 10C and discharge rate of 1C, respectively. c) Contour plots of in situ XRD characterization for P‐0.05 at a charge rate of 10C and discharge rate of 1C. d) The corresponding evolution of refined parameters (lattice volume and lattice parameter *c*) for the entire pattern of (c) and corresponding GCD curves. e) Contour plots of in situ XRD characterization for P‐0.05 at a charged/discharged rate of 10C. f) The corresponding evolution of refined parameters (lattice volume and lattice parameter c) for the entire pattern of (e) and corresponding GCD curves.

Leveraging the optimized sodium ion diffusion pathways for exceptional dynamic diffusion and the ultralow structural strain from the pinning effect of Na‐layer, P‐0.05 shows significant potential for enabling rapid‐charging and slow‐discharging capability, highly attractive for the commercialization of SIBs. To evaluate its performance under various charge–discharge rates, its cyclic behavior was systematically examined. As shown in **Figure** **6**a, despite the charging current being 50 times the discharging current (a charge rate of 5C and a discharge rate of 0.1C), the Coulombic efficiency (the ratio of discharge capacity to charge capacity) remained ≈100%, with negligible capacity fade after 100 cycles. Further, the cycling performance of P‐0.05 was tested under two different regimes: a charging rate of 5C and a discharging rate of 1C, and a charging rate of 10C with a discharging rate of 1C, as depicted in Figure 6b. In both cases, the material demonstrated ≈ c100% Coulombic efficiency and almost no decline in capacity retention after 200 cycles. As for P‐0, the mismatch between charging and discharging capacities during rapid‐charge and slow‐discharge processes leads to more Na^+^ being intercalated into the cathode than is extracted (Figure S12, Supporting Information). This results in the accumulation of Na^+^ at the cathode, forming an amorphous phase that further causes a sharp decrease in capacity.

Given the outstanding capability of P‐0.05 for rapid‐charging and slow‐discharging, this study, utilizing synchrotron radiation sources, delved into the structural evolution mechanism of P‐0.05 during such electrochemical processes. Figure 6c presents contour plots from in situ synchrotron XRD over five cycles (charging at 10C and discharging at 1C), where the evolution of diffraction peaks consistently showed typical solid‐solution behavior. Figure 6d shows the results from Rietveld refinement based on diffraction data from Figure 6c. Over five cycles, the maximum volume strain was only 0.12%, and the maximum strain along the *c*‐axis was merely 0.27%. Furthermore, in situ, XRD experiments were conducted on this sample under a 10C charge–discharge rate (Figure 6e), with refinement results indicating ultralow structural strain during the ten‐cycle process at 10C (maximum volume strain of 0.09% and maximum *c*‐axis strain of 0.14%). This ultralow strain property ensures the stability of its layered structure under different rates, forming the cornerstone of its exceptional rapid charging and slow discharging performance.

To further investigate the rapid‐charging and slow‐discharging capability of P‐0.05 in practical applications, it was assembled into a full cell with a hard carbon anode for asymmetric electrochemical testing. As shown in Figure S13a,b (Supporting Information), the GCD curves of the P‐0.05 cathode and hard carbon anode at 0.1C were used to match the capacity (N/P radio = 3.12, with a 10% excess of hard carbon to compensate for capacity loss at high rate), resulting in the assembly of a P‐0.05 || hard carbon full cell. As illustrated in Figure S13c (Supporting Information), the assembled full cell maintained excellent cycling stability during asymmetric electrochemical cycling at 5C charge and 1C discharge, retaining 94.4% of its capacity after 100 cycles.

**Figure 7:**
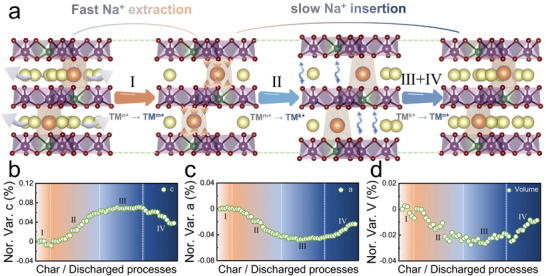
a) The schematic diagram of the crystal structure evolution of P‐0.05 during rapid charging (10C) and slow discharging (1C) processes. b–d) The corresponding evolution of refined lattice parameters b) *c*, c) *a*, and d) lattice volume during the second charged–discharged processes at the charge rate of 10C and discharge rate of 1C.

**Figure** **7**a presents a schematic representation illustrating the structural evolution of the P‐0.05 lattice structure along the [110] axis during the fast charging and slow discharging processes, with a specific focus on the changes in the *c*‐axis. Initially, due to the rivet effect of potassium ions and the excellent sodium ion diffusion kinetics of P‐0.05, along with the oxidation of TM ions partially shields the O–O repulsion between adjacent transition metal layers, thereby preventing significant expansion along the *c*‐axis during the rapid extraction of sodium ions (region 1 in Figure 7b). Additionally, owing to the delayed lattice expansion, the evolution of the *a*‐axis is minimal (Figure 7c), contrasting with the lattice evolution observed at a low rate (Figure 5c).

Moreover, during the low‐rate sodium insertion process, abnormal expansion along the *c*‐axis and negative expansion along the *a*‐axis occur. This phenomenon arises from the reduction of TM ions, which suppresses the shielding effect of O–O repulsion between adjacent transition metal layers during the charging process and releases sluggish lattice expansion kinetics. Subsequently, a competition mechanism arises between these abnormal phenomena and the factors inducing contraction along the *c*‐axis and expansion along the *a*‐axis in the conventional sodium insertion process (Figure 5c). As a result, during the discharging process, both parameters *c* and *a* undergo conventional contraction and expansion, respectively, after a plateau phase.

Due to this unique lattice evolution, the maximum structural evolution of P‐0.05 throughout the entire charge–discharge process is only 0.03% (Figure 7d), demonstrating excellent reproducibility in subsequent cycles (Figure S14, Supporting Information). This indicates that the rivet effect of sodium layers and the regulation of sodium ion diffusion pathways not only optimize the sodium ion diffusion kinetics but also confer excellent structural stability to the layered cathode, enabling P‐0.05 to achieve stable and reversible fast charging and slow discharging performance. Furthermore, during fast charging and discharging processes, the maximum structural strain per cycle of P‐0.05 is only 0.003% (Figure 6f), underscoring its promising prospects as an advanced sodium cathode material. As shown in Table S4 (Supporting Information), a comprehensive performance comparison is conducted between the P‐0.05 synthesized in this work and the currently reported near‐zero strain SIBs cathode materials. P‐0.05 exhibits significant advantages in rate performance while maintaining ultralow volume strain, endowing it with excellent rapid‐charging and slow‐discharging capacity.

## 4 Conclusion

In summary, this study addresses the optimization of sodium layer doping in P2‐phase layered oxide materials by introducing potassium ions with high migration barriers and interlayer anchoring effects into the common Na_0.67_Ni_0.33_Mn_0.67_O_2_. This modification optimizes the sodium ion diffusion pathway, significantly improving sodium ion diffusion kinetics and increasing the sodium ion diffusion rate by an order of magnitude. Consequently, the modified material achieves 94% of its capacity expression at a 0.1C rate under a 20C rate. Furthermore, the rivet effect of potassium ions results in a volume evolution rate of only 0.03% during the asymmetric electrochemical process of 10C charging and 1C discharging, representing a truly zero‐strain cathode. Moreover, under various asymmetric rates, the modified samples exhibit almost negligible capacity decay over 200 cycles. These advancements in sodium ion diffusion kinetics and structural stability contribute to the superior rapid‐charging and slow‐discharging performance of the SIB cathode, thus opening up new avenues for its commercialization process.

## 5 Experimental Section

### Materials Synthesis

The Na_0.67_Ni_0.33_Mn_0.67_O_2_ (P‐0), Na_0.62_K_0.05_Ni_0.33_Mn_0.67_O_2_ (P‐0.05), and Na_0.57_K_0.10_Ni_0.33_Mn_0.67_O_2_ (P‐0.10) used in this paper were synthesized via a simple solid‐state method. In the typical synthesis procedure, stoichiometric values of Na_2_CO_3_ (with a 5% excess, Sinopharm Chemical, 99.8%), K_2_CO_3_ (Sinopharm Chemical, 99%), NiO (Aladdin, 99.9%) and Mn_2_O_3_ (98%, Sinopharm Chemical) were meticulously mixed in the high‐energy ball mill machine (MSK‐SFM‐1S). The resulting mixture was then pressed into pellets, which were subsequently heated at 900 °C for 16 h within an air atmosphere. The as‐prepared materials were promptly transferred into an inert Ar atmosphere to prevent exposure to air and were stored for subsequent characterization.

### Electrochemical Measurement

All electrochemical performances of the cathodes were systematically examined in sodium half‐cells. For the preparation of the working electrode, a slurry was created, comprising 80 wt.% active material, 10 wt.% poly(vinylidene fluoride) (PVDF, with 5 wt.% Solvay 5130 PVDF binder dissolved in N‐methyl‐2‐pyrrolidone (NMP)), 10 wt.% acetylene black, and a suitable quantity of NMP. This mixture was uniformly dispersed onto a 10 µm thick aluminum foil. After drying in an oven at 90 °C, the electrodes were cut into 10 mm diameter disks and further dried at 110 °C under vacuum for 12 h to eliminate all solvent traces.

Pure sodium foil served as the counter electrode, and glass fiber (GB‐100R, ADVANTEC Co., Japan) was utilized as the separator. The electrolyte consisted of 1 m NaClO_4_ dissolved in a nonaqueous solution of ethylene carbonate (EC)/diethyl carbonate (DEC) with equal volumes and 5% fluoroethylene carbonate (FEC). The assembly of coin‐type half‐cells (2032) was conducted in an argon‐filled glove box (Mikrouna Super 1220) with H_2_O and O_2_ content maintained below 0.01 ppm.

Typical galvanostatic discharge/charge tests were performed on a Neware Battery Tester within a voltage window of 2.0–4.1 V. GITT measurements were conducted using a Maccor test cabinet, while CV and EIS tests were carried out with an OCTOSTAT200 electrochemical workstation (IVIUM Instrument).

GITT measurements were conducted by periodically pulsing and relaxing the battery within the voltage range of 2.0–4.1 V using a NEWARE electrochemical analyzer. Each step consisted of a 15 min pulse at 15 mA g^−1^, followed by a 90 min relaxation period. The diffusion coefficient of Na^+^ (D_Na_^+^) was calculated using Equation (1):^[^ ^20^ ^]^

(1) $$D_{Na^{+}}=\frac{4}{\pi \tau }{\left(\frac{m_{B}V_{M}}{M_{B}A}\right)}^{2}{\left(\frac{\Delta E_{s}}{\Delta E_{\tau }}\right)}^{2}$$

Where *τ* is the duration time of the current pulse, *m_B_* is the mass of the active material (g), *V_M_* is the molar volume (cm^3^ mol^−1^), *M_B_* is the molecular weight (g mol^−1^), *A* is the total contact area between electrode and electrolyte (cm^2^), *ΔE_τ_* is the variation of the cell voltage, *ΔE*_s_ is related to the change of steady‐state voltage for the corresponding step. Equation (1) provides a method for calculating the diffusion coefficient of Na^+^ based on the given parameters.

### Structural Characterization

The crystalline structures of the samples were investigated by both X‐ray and neutron diffraction measurements. The X‐ray diffraction (XRD) patterns of the powder samples were collected by Bruker D8 Discover diffractometer with Cu Kα radiation at room temperature. The neutron powder diffraction (NPD) experiment was carried out on General Purpose Powder Diffractometer (GPPD), a time‐of‐flight (TOF) diffractometer at China Spallation Neutron Source (CSNS), Dongguan, China. Then the Fullprof program was used to analyze crystal structure by the Rietveld refinement methods.^[^ ^23^ ^]^ As for in situ XRD studies at the rate of 0.1C, the experiment was carried out on the same XRD diffractometer combined with an in situ device. In situ XRD tests at 10C charging/1C discharging and 10C charging/10C discharging were performed with Liquid Metal Jet Source (Ga Kɑ: 9.24 keV, wavelength: 1.3418 Å) at the Institute of Advanced Science Facilities, Shenzhen with Dectris PILATUS 1 M detector in the 2θ range of 10°–50°.

The morphology and element distribution of the samples were investigated by a scanning electron microscope (SEM, ZEISS SUPRA 55) with an EDS (OXFORD, X‐MaxN TSR).

### Synchrotron X‐Ray Absorption Spectroscopy Measurements

The electrodes were dissembled from the coin cells charged or discharged to different states. With high penetration and low absorption, Synchrotron X‐ray precisely reflected bulk sample structure properties, which was beneficial when observing tiny phase changes that were usually invisible in laboratory‐scale X‐ray diffraction due to poor background noise. Hard X‐ray absorption spectroscopy (hXAS) of the samples was conducted at the BL11B beamline of Shanghai Synchrotron Radiation Facility (SSRF), Shanghai, China.

### TEM and STEM Measurements

The TEM and HRTEM were conducted through a transmission electron microscope (TEM, JEM–3200FS, 300 keV) with an EDS (OXFORD, X‐MaxN TSR). Additionally, electron microscopy including HAADF imaging, ABF imaging, EDS, and EELS was conducted on a Cs‐corrected transmission electron microscope (Titan Cubed Themis Z 60–300 kV, Thermo Fisher Scientific) operated at 300 kV.

## Conflict of Interest

The authors declare no conflict of interest.

## Author Contributions

M.Y. and Z.C. contributed equally to this work. M.Y., Z.C., T.Y., and Y.X. conceived the idea and designed the experiments. M.Y. and Z. Chen. synthesized all the materials and conducted electrochemical measurements. M.Y., Z.C., Z.H., R.W., and D.Z. carried out the in situ XRD and NPD. T.Y. and M.Y. carried out the SEM, TEM, EELS, and STEM measurements and data analysis. M.Y., Z.C. W.J., and T.Z. performed ex situ synchrotron XAS. M.Y., Z.C., T.Y., and Y.X. wrote the manuscript and all authors edited the manuscript.

## Supporting information

Supporting Information

## Data Availability

The data that support the findings of this study are available from the corresponding author upon reasonable request.
