# Supplementary material for: Layered Cathode with Ultralow Strain Empowers Rapid‐Charging and Slow‐Discharging Capability in Sodium Ion Battery
Source: Adv Sci (Weinh). 2024 Jun 28;11(33):2404701. doi: 10.1002/advs.202404701 (PMC11434015; doi:10.1002/advs.202404701)
Supplement: Supplementary file 1 — Supporting Information [file ADVS-11-2404701-s001.docx]

Supporting Information

**Layered Cathode with Ultralow Strain Empowers Rapid-charging and Slow-Discharging Capability in Sodium Ion Battery**

*Maolin Yang, Ziwei Chen, Zhongyuan Huang, Rui Wang, Wenhai Ji, Dong Zhou, Tao Zeng, Yongsheng Li, Jun Wang, Liguang Wang, Tingting Yang,^*^ and Yinguo Xiao^*^*

M. Yang, Z. Chen, Z. Huang, T. Zeng, Y. Li, T. Yang, Y. Xiao

School of Advanced Materials, Peking University, Shenzhen Graduate School, Shenzhen 518055, PR China

E-mail: t.yang@fz-juelich.de, y.xiao@pku.edu.cn

T. Yang

Ernst Ruska-Centre for Microscopy and Spectroscopy with Electrons, Forschungszentrum Jülich GmbH, Jülich 52428, Germany

R. Wang

Department of Engineering, University of Cambridge, Cambridge CB30FS, UK

W. Ji

Spallation Neutron Source Science Center, Dongguan 523803, China

D. Zhou

School of Advanced Energy, Shenzhen Campus of Sun Yat-sen University, Shenzhen 518107, P. R. China

J. Wang

School of Innovation and Entrepreneurship, Southern University of Science and Technology, Shenzhen 518055, PR China

L. Wang

College of Chemical and Biological Engineering, Zhejiang University, Hangzhou 310000, China

**Table S1. The Rietveld refinement result of neutron powder diffraction for P-0.**

| P-0 (97.05 wt%) – *P6_3_* | | | | | | |
| --- | --- | --- | --- | --- | --- | --- |
| Atom | Wyckoff | x | y | z | Biso | Occ |
| O | 6c1 | 0.354(5) | 0.331(3) | 0.407(7) | 0.548(4) | 1 |
| O | 6c2 | 0.698(4) | 0.679(0) | -0. 407(7) | 0.548(4) | 1 |
| Na | 6c | 0.758(8) | 0.716(4) | 0.263(5) | 1.818(4) | 0.439(8) |
| Na | 2a | 0 | 0 | 0.263(5) | 1.868(2) | 0.076(7) |
| Na | 2b | 1/3 | 2/3 | 0.263(5) | 1.868(2) | 0.076(7) |
| Na | 2b | 2/3 | 1/3 | 0.263(5) | 1.868(2) | 0.076(7) |
| Mn | 2a1 | 0 | 0 | 0 | 0.032(3) | 0.286(3) |
| Ni | 2a1 | 0 | 0 | 0 | 0.032(3) | 0.047(1) |
| Mn | 2b2 | 2/3 | 1/3 | 0 | 0.032(3) | 0.094(2) |
| Ni | 2b2 | 2/3 | 1/3 | 0 | 0.032(3) | 0.239(1) |
| Mn | 2b3 | 1/3 | 2/3 | 0 | 0.032(3) | 0.286(3) |
| Ni | 2b3 | 1/3 | 2/3 | 0 | 0.032(3) | 0.047(1) |
| *a* = *b* = 4.994(7) Å, *c* = 11.133(2) Å, V = 240.530 Å^3^, α = β = 90°, γ = 120°  Rp = 5.52, Rwp = 8.19, Rexp = 2.56 | | | | | | |

**Table S2. The Rietveld refinement result of neutron powder diffraction for P-0.05.**

| P-0.05 (97.55 wt%) – *P6_3_* | | | | | | | |
| --- | --- | --- | --- | --- | --- | --- | --- |
| Atom | Wyckoff | x | y | | z | Biso | Occ |
| O | 6c1 | 0.358(3) | 0.331(5) | 0.407(9) | | 0.896(9) | 1 |
| O | 6c2 | 0.699(9) | 0.686(6) | -0.407(9) | | 0.896(9) | 1 |
| Na | 6c | 0.703(8) | 0.756(1) | 0.243(8) | | 2.546(2) | 0.389(6) |
| K | 6c | 0.703(8) | 0.756(1) | 0.243(8) | | 2.546(2) | 0.050(0) |
| Na | 2a | 0 | 0 | 0.243(8) | | 2.546(2) | 0.076(8) |
| Na | 2b | 1/3 | 2/3 | 0.243(8) | | 2.546(2) | 0.076(8) |
| Na | 2b | 2/3 | 1/3 | 0.243(8) | | 2.595(6) | 0.076 (8) |
| Mn | 2a1 | 0 | 0 | 0 | | 0.017(1) | 0.271(1) |
| Ni | 2a1 | 0 | 0 | 0 | | 0.017(1) | 0.062(2) |
| Mn | 2b2 | 2/3 | 1/3 | 0 | | 0.017(1) | 0.124(4) |
| Ni | 2b2 | 2/3 | 1/3 | 0 | | 0.017(1) | 0.208(9) |
| Mn | 2b3 | 1/3 | 2/3 | 0 | | 0.017(1) | 0.271(1) |
| Ni | 2b3 | 1/3 | 2/3 | 0 | | 0.017(1) | 0.062(2) |
| *a* = *b* = 4.995(0) Å, *c* = 11.135(9) Å, V = 240.613 Å^3^, α = β = 90°, γ = 120°  Rp = 5.87, Rwp = 8.17, Rexp = 2.73 | | | | | | | |

**Table S3. The Rietveld refinement result of neutron powder diffraction for P-0.10.**

| P-0.10 (95.64 wt%) – *P6_3_* | | | | | | | |
| --- | --- | --- | --- | --- | --- | --- | --- |
| Atom | Wyckoff | x | y | | z | Biso | Occ |
| O | 6c1 | 0.355(6) | 0.333(7) | 0.407(9) | | 0.495(6) | 1 |
| O | 6c2 | 0.694(8) | 0.681(5) | -0.407(9) | | 0.495(6) | 1 |
| Na | 6c | 0.709(1) | 0.745(3) | 0.244(1) | | 2.548(8) | 0.322(2) |
| K | 6c | 0.709(1) | 0.745(3) | 0.244(1) | | 2.548(8) | 0.100(1) |
| Na | 2a | 0 | 0 | 0.244(1) | | 2.598(4) | 0.082(6) |
| Na | 2b | 1/3 | 2/3 | 0.244(1) | | 2.598(4) | 0.082(6) |
| Na | 2b | 2/3 | 1/3 | 0.244(1) | | 2.598(4) | 0.082(6) |
| Mn | 2a1 | 0 | 0 | 0 | | 0.032(2) | 0.289(6) |
| Ni | 2a1 | 0 | 0 | 0 | | 0.032(2) | 0.043(7) |
| Mn | 2b2 | 2/3 | 1/3 | 0 | | 0.032(2) | 0.087(3) |
| Ni | 2b2 | 2/3 | 1/3 | 0 | | 0.032(2) | 0.246(1) |
| Mn | 2b3 | 1/3 | 2/3 | 0 | | 0.032(2) | 0.289(6) |
| Ni | 2b3 | 1/3 | 2/3 | 0 | | 0.032(2) | 0.043(7) |
| *a* = *b* = 4.994(3) Å, *c* = 11.139(1) Å, *V* = 240.612 Å^3^, α = β = 90°, γ = 120°  Rp = 4.89, Rwp = 7.04, Rexp = 2.66 | | | | | | | |

**Table S4. Comparison of comprehensive performance with reported nearly zero-strain SIBs cathodes.**

| Sample | rate_Max_/rate_min_ (Capacity retention) | Specific capacity (mAh g^-1^)^*^ | V  variation | Ref |
| --- | --- | --- | --- | --- |
| Na_0.80_Li_0.024_[Li_0.065_Ni_0.22_Mn_0.66_]O_2_ | 10C/0.1C (87%) | 92 | 0.7% | [S1] |
| Na_0.75_Li_0.15_Mg_0.05_Ni_0.1_Mn_0.7_O_2_ | 5C/0.2C (66.6%) | 125.7 | 0.49 | [S2] |
| Na_0.85_Li_0.12_Ni_0.22_Mn_0.66_O_2_ | 20C/0.1C (64.3%) | 123.4 | 1.7% | [S3] |
| Na_0.67_Mn_0.7_Zn_0.15_Mg_0.15_O_2_ | 10C/0.05C (40.4%) | ~90.3 | 0.55% | [S4] |
| Na_0.67_Mn_0.5_Co_0.4_Fe_0.1_O_2_ | 10C/0.2C (~60%) | ~140 | 0.6% | [S5] |
| Na_0.65_Li_0.08_Cu_0.08_Fe_0.24_Mn_0.6_O_2_ | 5C/0.1C (76%) | 85 | 0.7% | [S6] |
| P3-Na_0.75_Mg_0.08_Co_0.10_Ni_0.2_Mn_0.60_O_2_ | 10C/0.2C (66.5%) | 120 | 1.4% | [S7] |
| O3-Na_0.73_Li_0.36_Ti_0.73_O_2_ | 1.8C/0.1C (69.4%) | ~108 | 1.88% | [S8] |
| P2/P3-NaNi_1/3_Co_1/3_Mn_1/3_O_2_ | 3C/0.2C (~64%) | 125.1 | 1.3%-P2, 45%-P3 | [S9] |
| Na_3_Mn_2_(P_2_O_7_)(PO_4_) | 5C/0.1C (31.7%) | ~90 | 0.87% | [S10] |
| Na_0.62_K_0.05_Ni_0.33_Mn_0.67_O_2_ (P-0.05) | 20C/0.1C (94%) | 90 ^**^ | 0.53% | This work |
|  |  | 87.5^***^ | 0.03% |  |
|  |  | 84.6^****^ | 0.003% |  |

Note:

* The discharge specific capacity during in-situ XRD testing;

** Electrochemical testing mode: 0.1C charging and discharging;

*** Electrochemical testing mode: 10C charging and 1C discharging;

**** Electrochemical testing mode: 10C charging and 10C discharging.


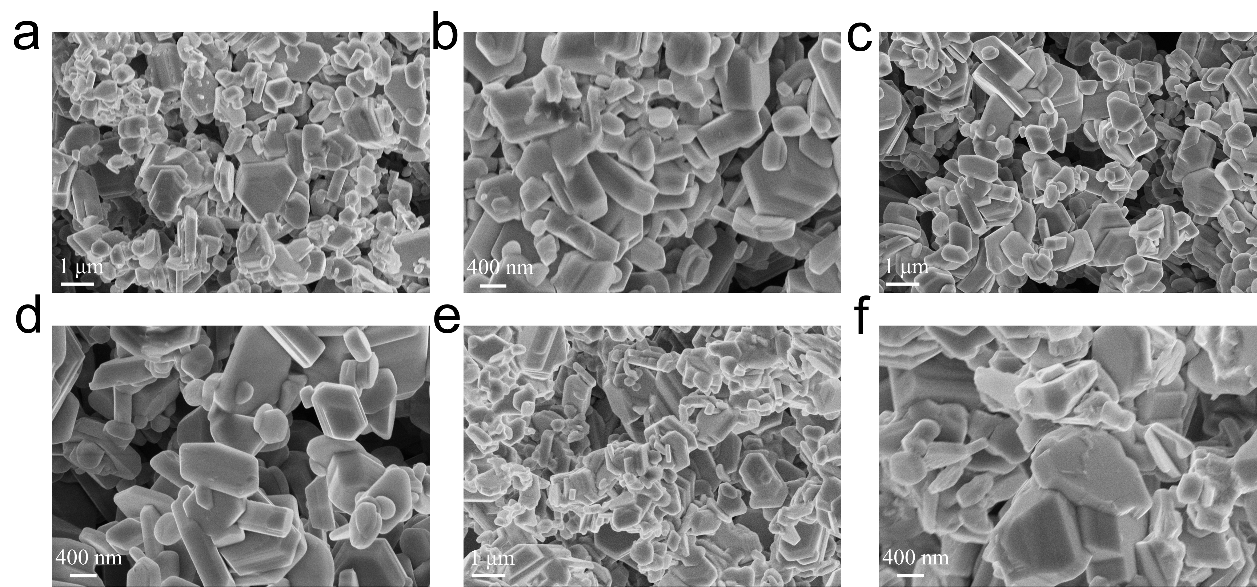


**Figure S1.** (a-f) SEM images of P-0 (a-b), P-0.05 (c-d) and P-0.10 (e-f) at different magnification scales.


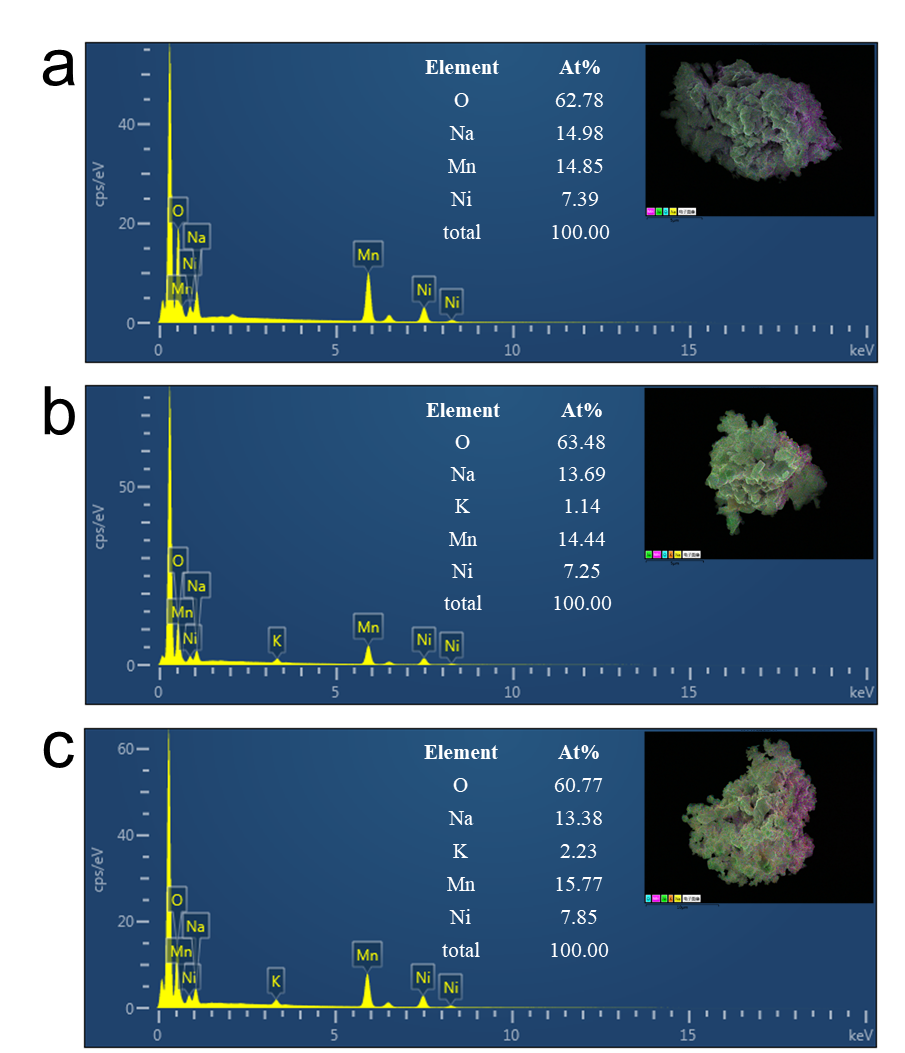


**Figure S2.** (a-c) The calculated atomic ratios of P-0 (a), P-0.05 (b) and P-0.10 (c) based on the EDS elemental mapping results.


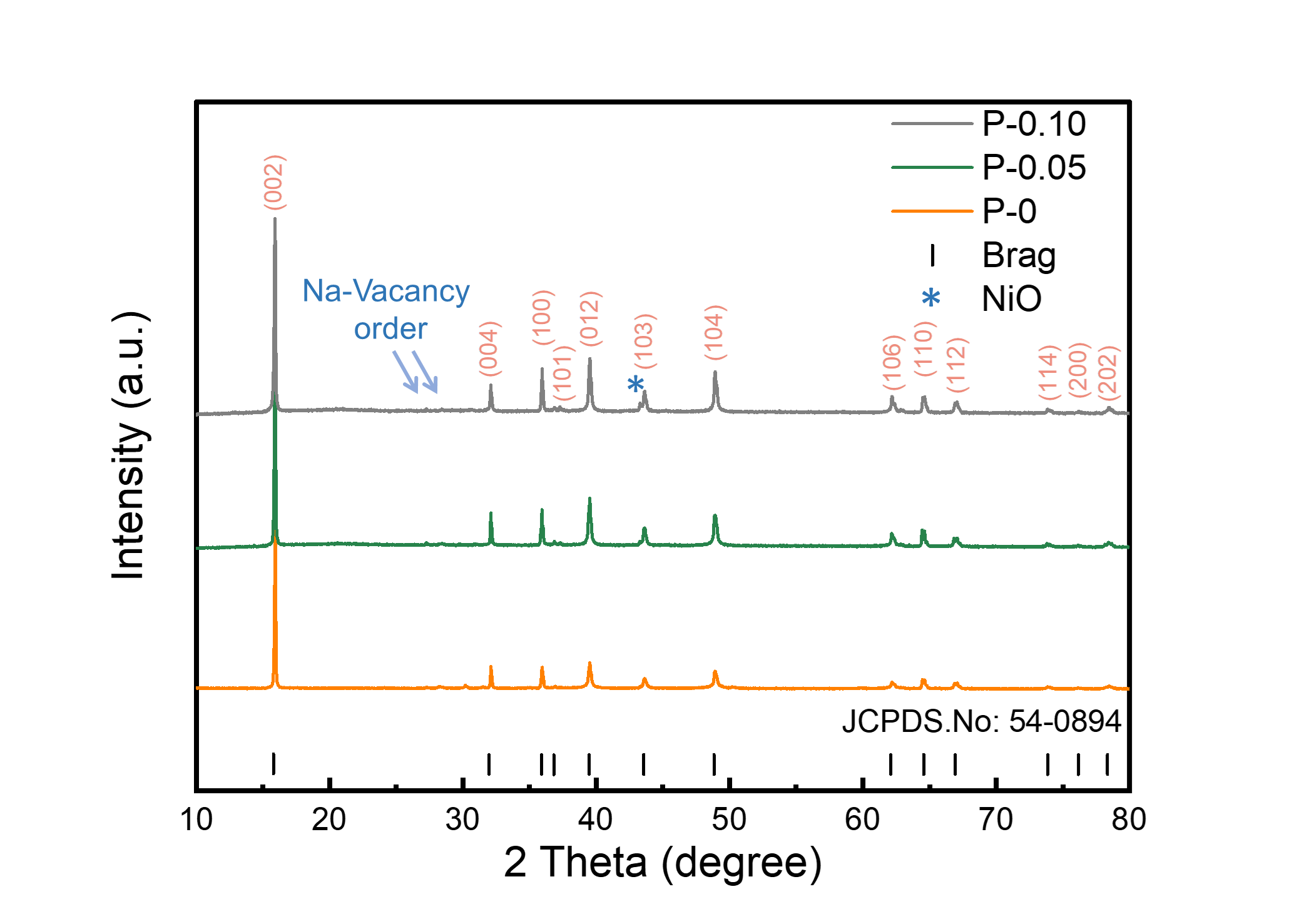


**Figure S3.** XRD patterns of P-0, P-0.05 and P-0.10.


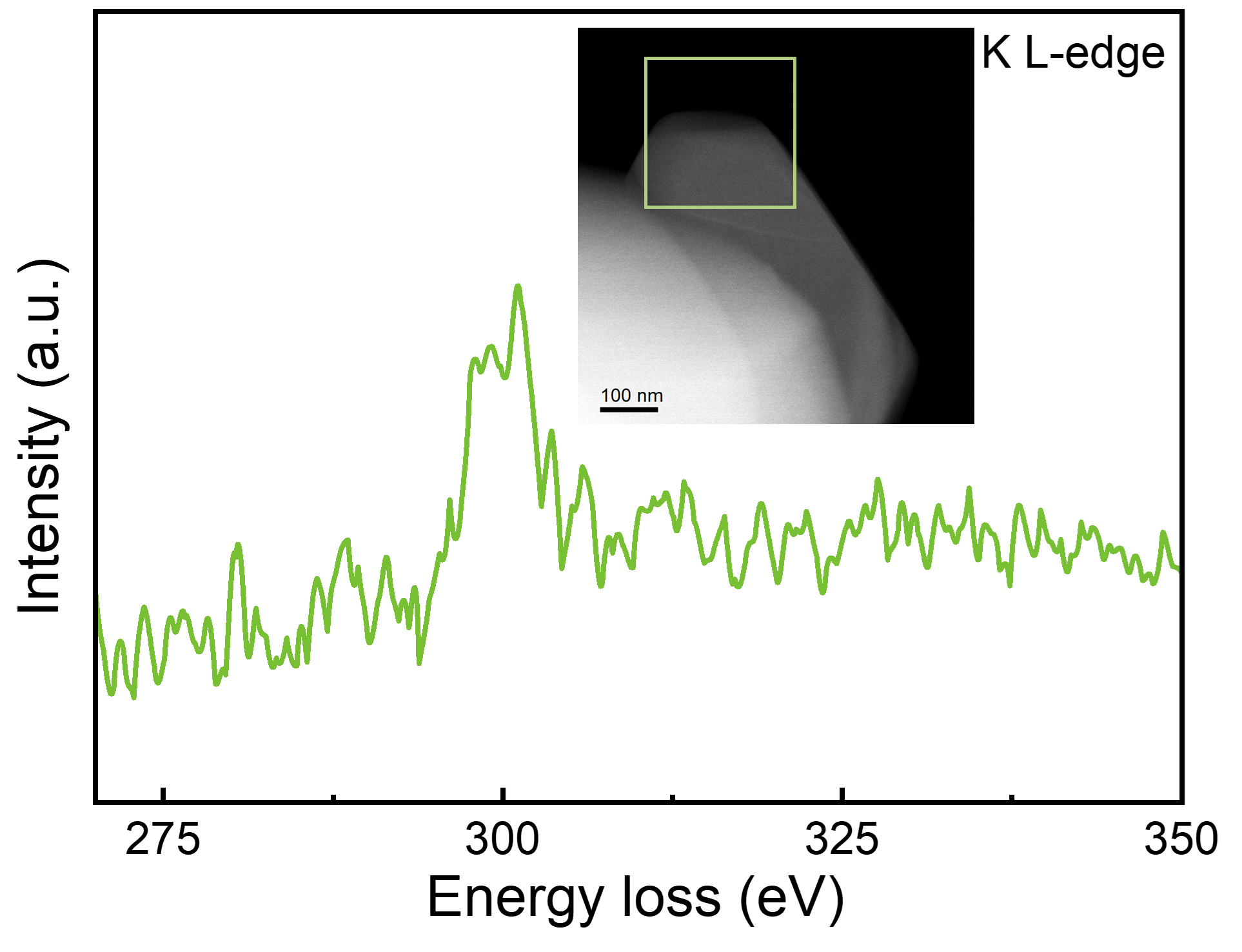


**Figure S4.** EELS spectrum of the selected area in the inset image.


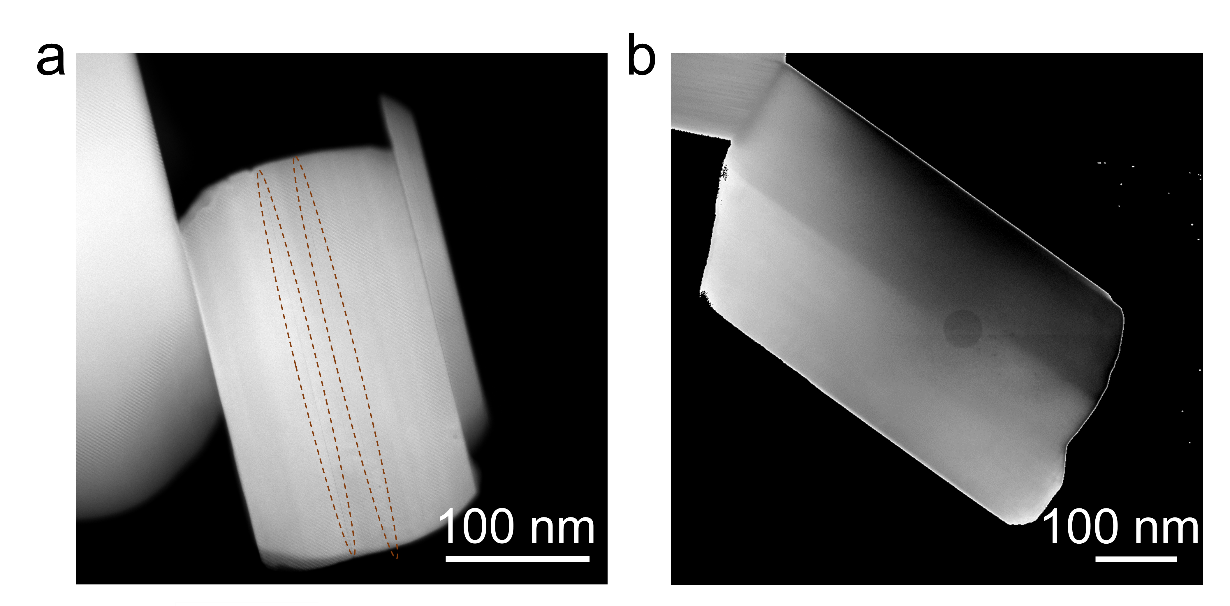


**Figure S5.** TEM images of P-0 (a) and P-0.05 (b) at the pristine state.


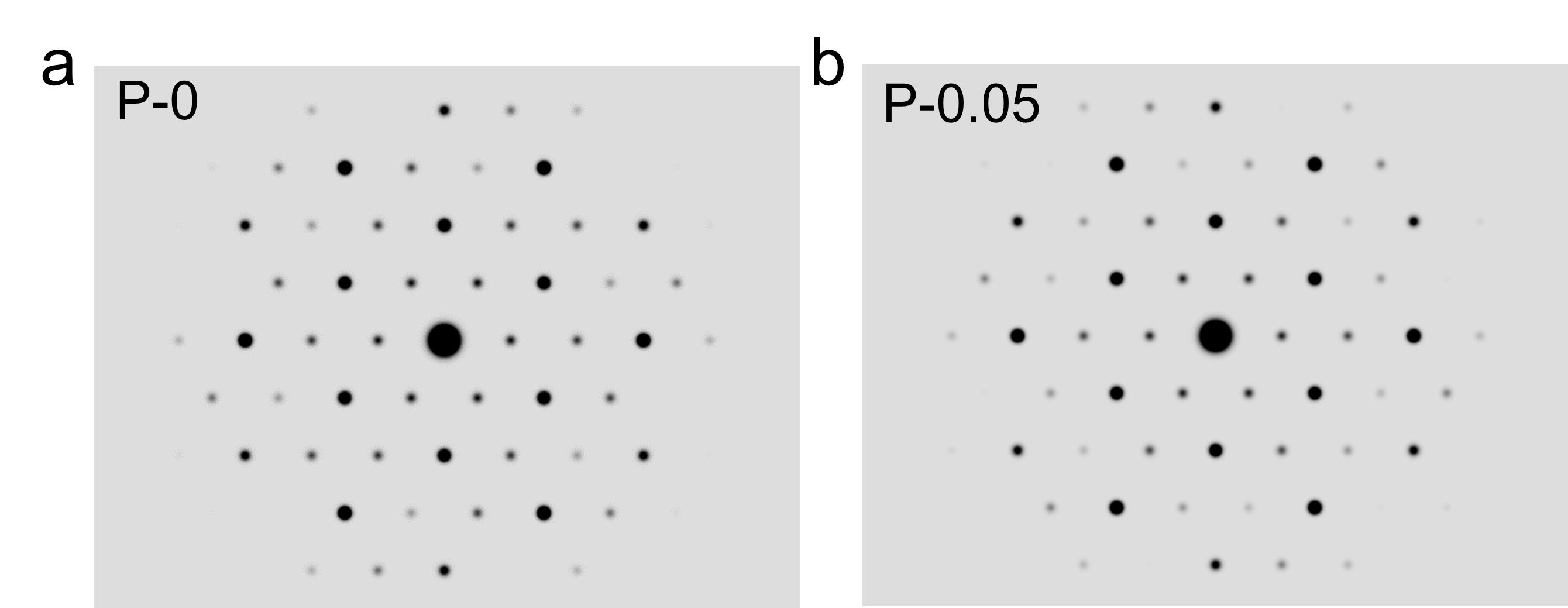


**Figure S6.** (**a-b**) Simulated electron diffraction patterns along the [001] zone axis of P-0 (**a**) and P-0.05 (**b**).


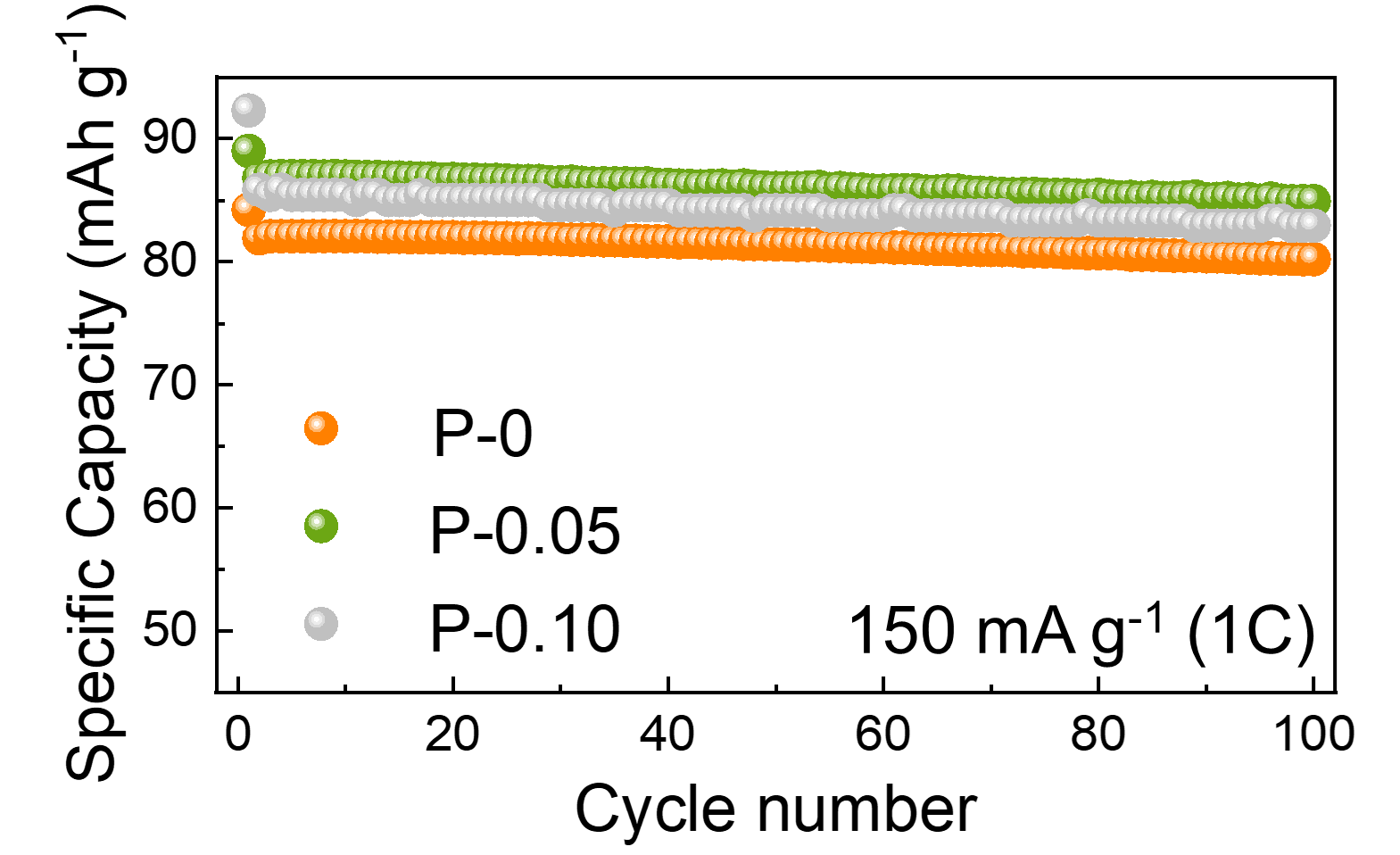


**Figure S7.** Long-term cycling retention of P-0, P-0.05 and P-0.10 at rates of 1C.


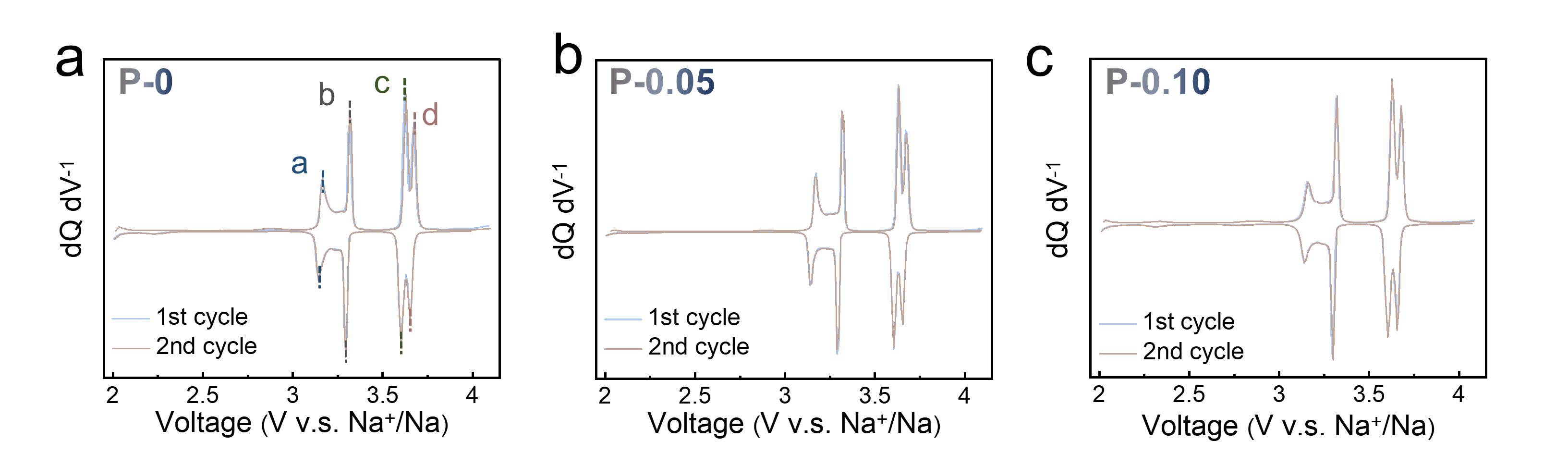


**Figure S8**. (a-c) dQ dV^-1^ curves of initial 2 cycles for P-0 (a), P-0.05 (b) and P-0.10 (c) at a rate of 0.1C.


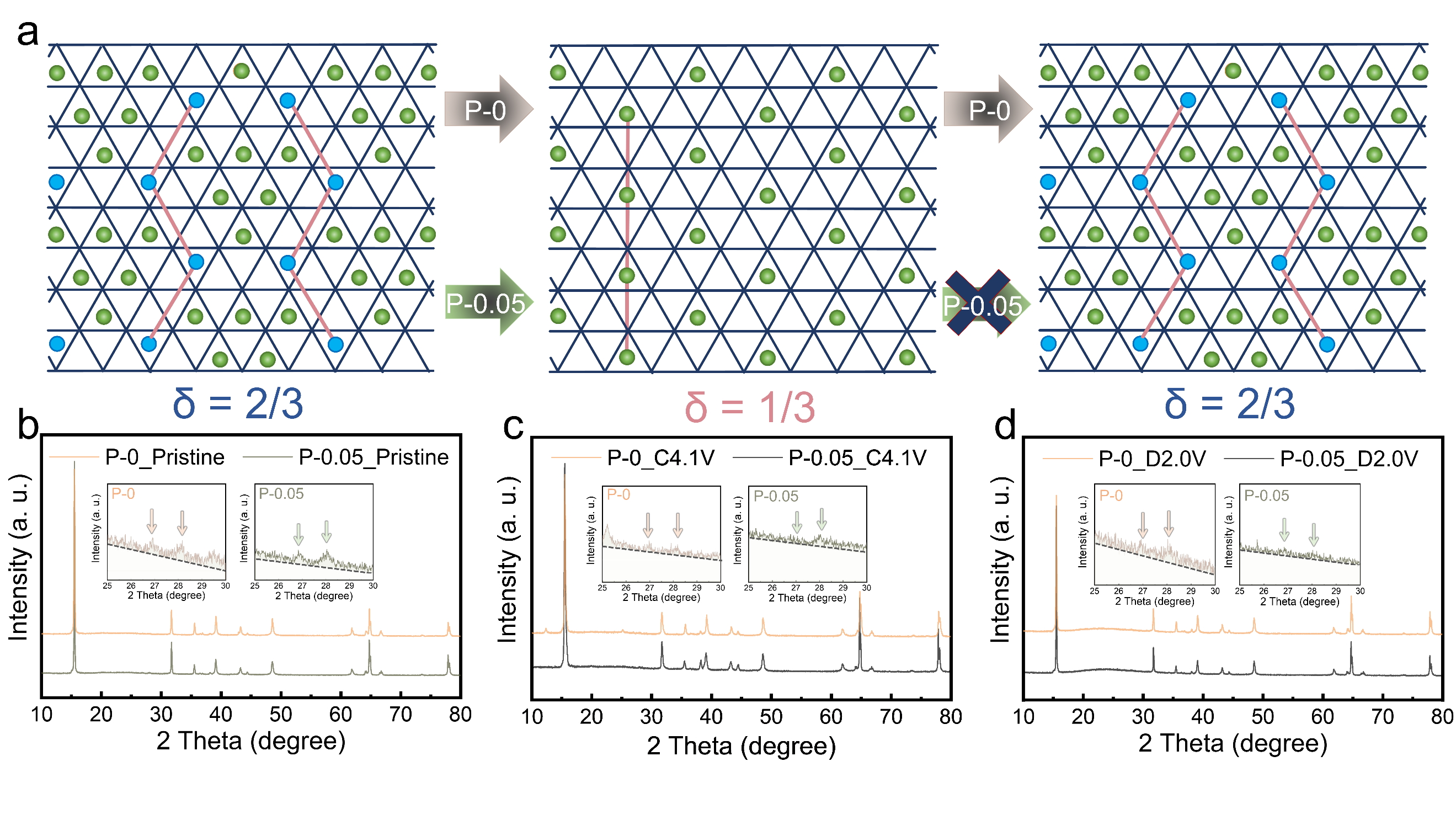


**Figure S9.** (a) Diagram illustrating the in-plane Na-ion orderings of Na_δ_[Ni_1/3_Mn_2/3_]O_2_ in the triangular lattice. From left to right: δ = 2/3, 1/2, and 1/3, respectively. (Blue balls represent Na ions on Nae sites, while green balls represent Na ions on Naf sites). (b-d) Ex-situ XRD patterns of P-0 and P-0.05 at pristine (b), 1st charged to 4.1V (c) and 1st discharged to 2.0V (d). (Insets show magnified patterns focused on the degree within 26-30°).


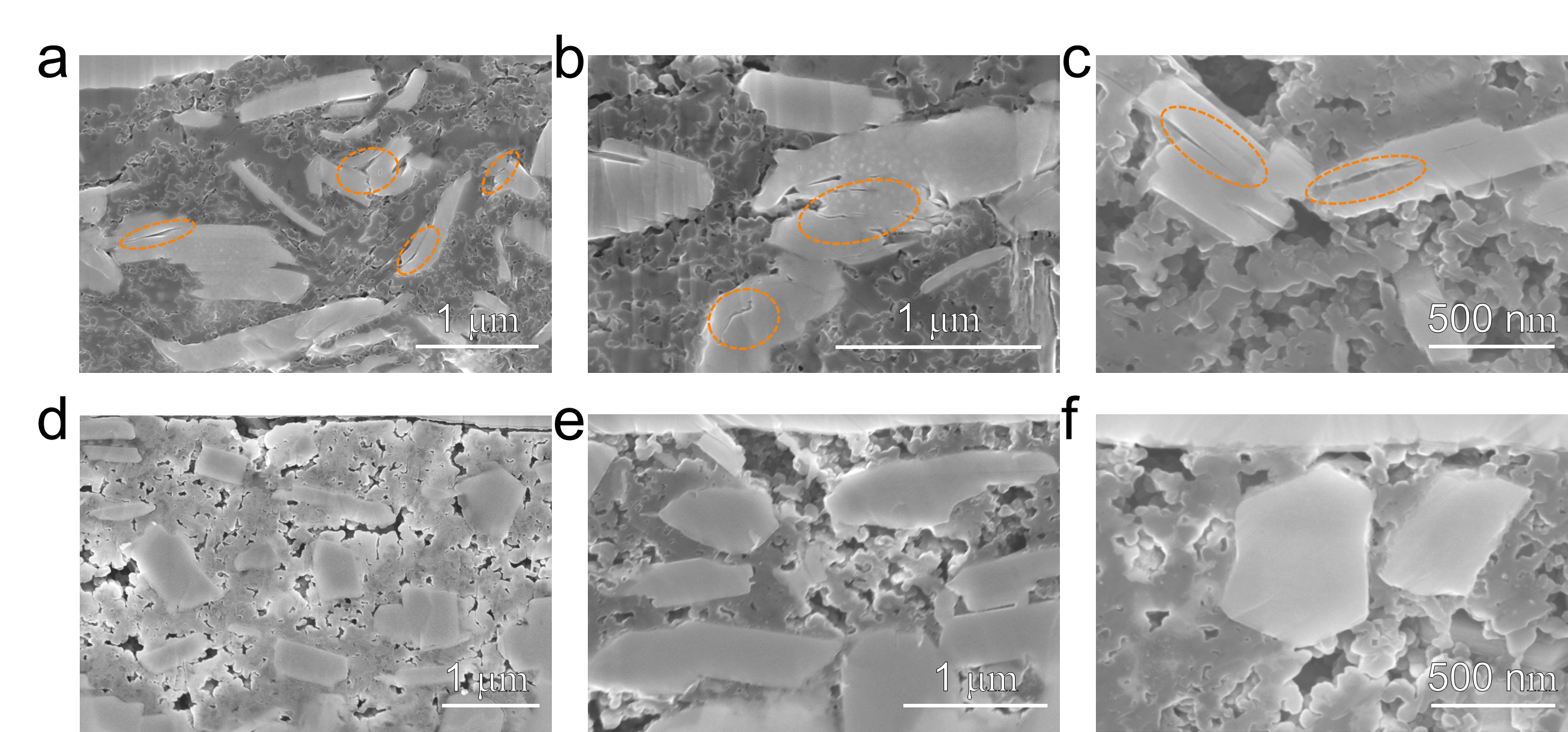


**Figure S10.** (a-f) Cross-sectional SEM images of the P-0 (a-c) and P-0.05 at different magnifications after 500 cycles at 10C.


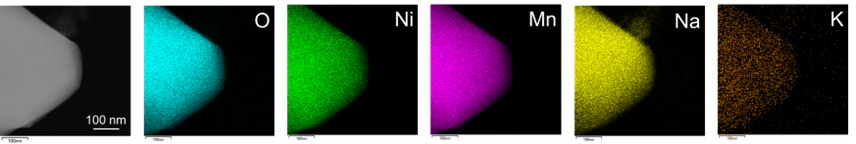


**Figure S11.** EDS elemental mapping of P-0.05 after 500 cycles at 10C.


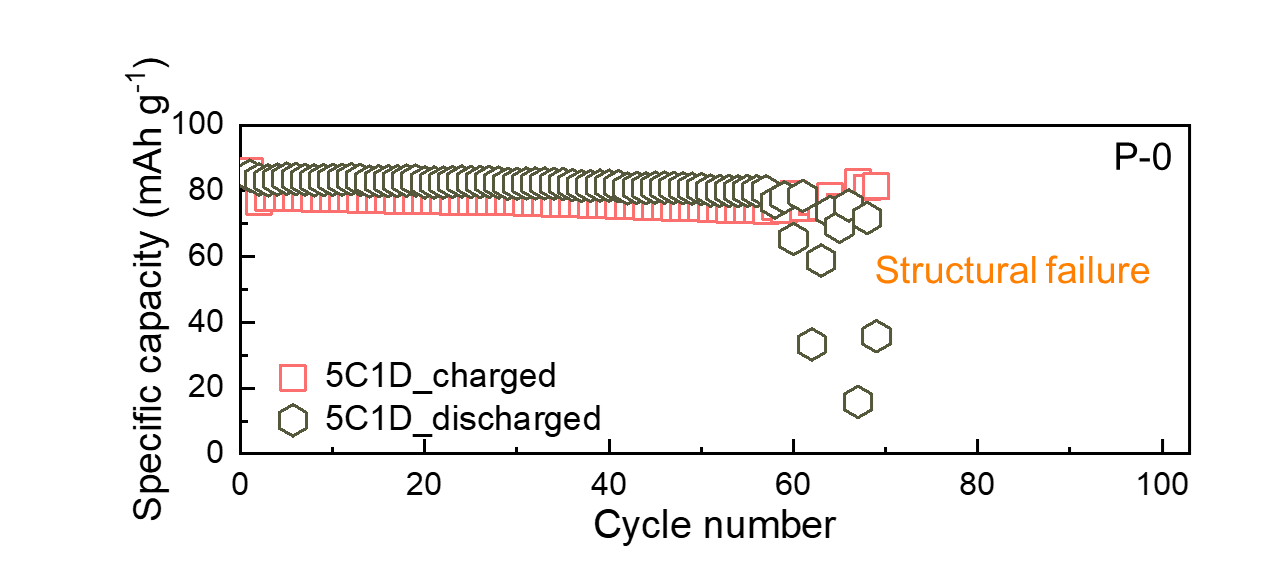


**Figure S12.** Long-term cycling retention of P-0 at charge rate of 5C and discharge rate of 1C.


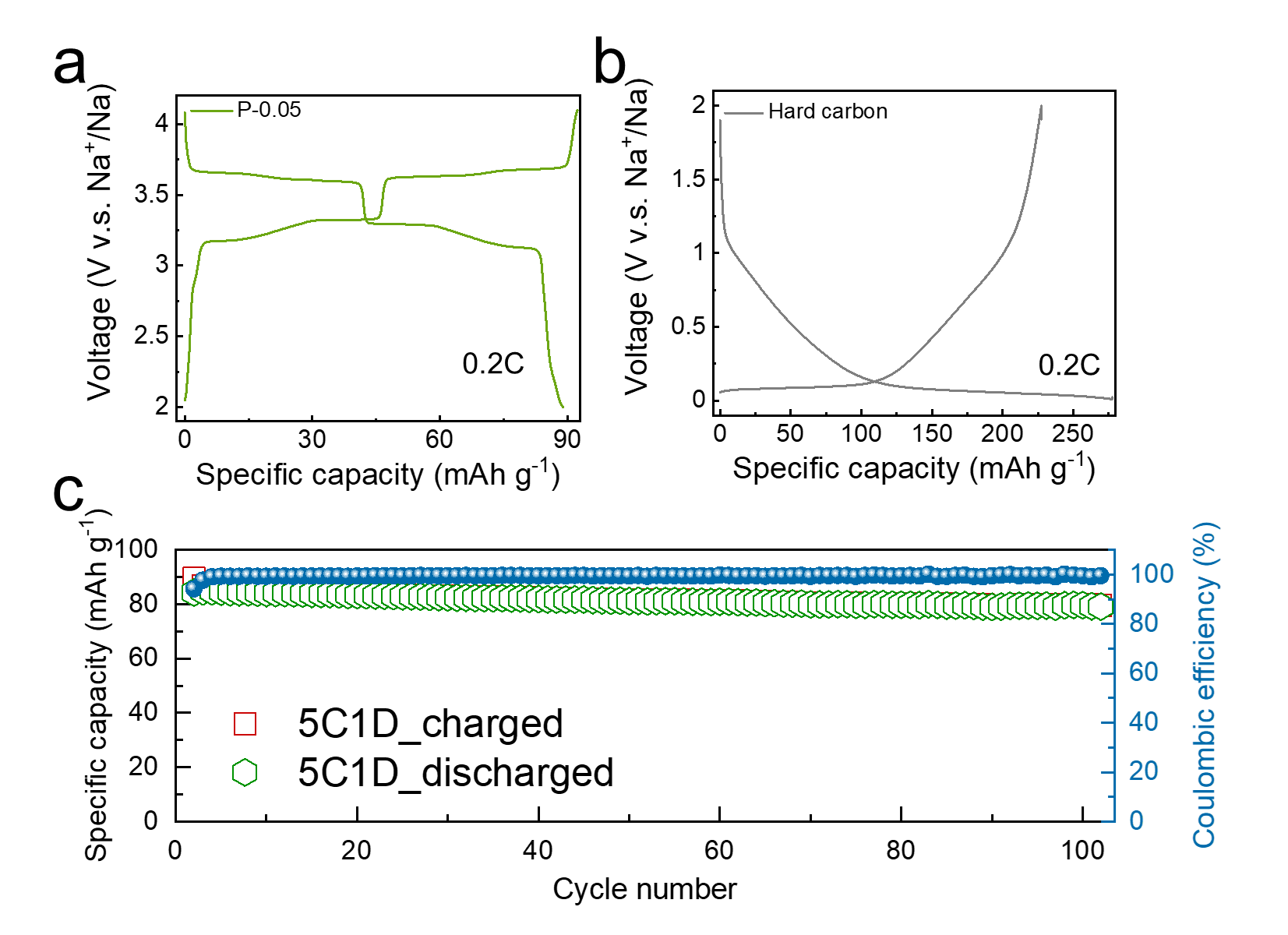


**Figure S13.** (a, b) The second cycle GCD curve of P-0.05 (a) and hard carbon (b) at 0.2C in half cell test within 2.0-4.1 V and 0.01-2.0 V, respectively. (c) Cycling performance of P-0.05 || hard carbon full cell at charge rate of 5C and discharge rate of 1C within 2.0-4.05 V.


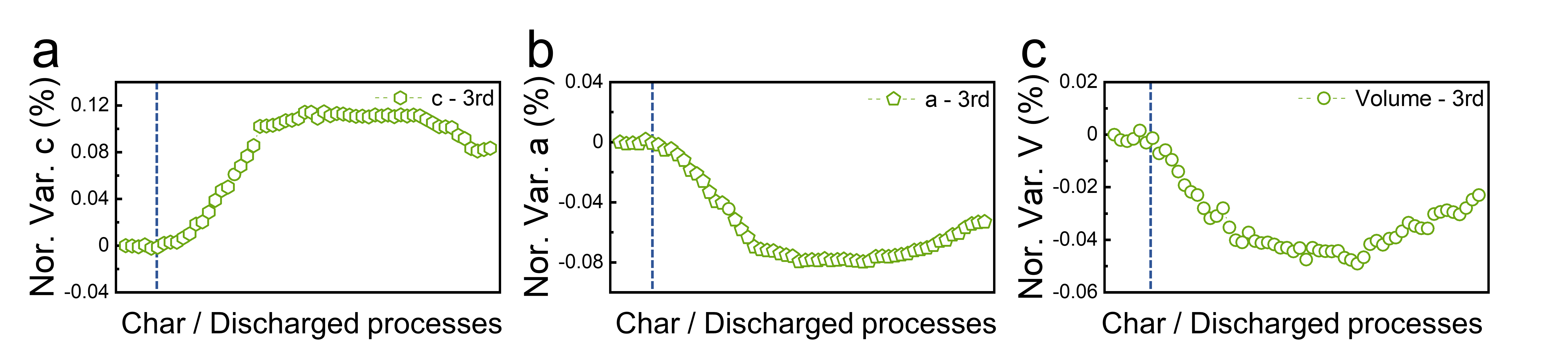


**Figure S14.** (a-c) The corresponding evolution of refined lattice parameters c (a), a (b) and lattice volume (c) during the third charged-discharged processes at the charge rate of 10 C and discharge rate of 1C. The dashed line in the figures separate the charging and discharge process.

**Reference**

[S1] X. Yang, S. Wang, H. Li, J. Peng, W.-J. Zeng, H.-J. Tsai, S.-F. Hung, S. Indris, F. Li, W. Hua, *ACS Nano* **2023**, 17, 18616.

[S2] M.-Y. Shen, J.-S. Wang, Z. Ren, T. Wu, X. Liu, L. Chen, W.-C. Li, A.-H. Lu, *Advanced Functional Materials* **2023**, 33, 2303812.

[S3] T. Jin, P.-F. Wang, Q.-C. Wang, K. Zhu, T. Deng, J. Zhang, W. Zhang, X.-Q. Yang, L. Jiao, C. Wang, *Angewandte Chemie International Edition* **2020**, 59, 14511.

[S4] X. Huang, D. Li, H. Huang, X. Jiang, Z. Yang, W. Zhang, *Nano Research* **2021**, 14, 3531.

[S5] S. Chu, C. Zhang, H. Xu, S. Guo, P. Wang, H. Zhou, *Angewandte Chemie International Edition* **2021**, 60, 13366.

[S6] R. Qi, M. Chu, W. Zhao, Z. Chen, L. Liao, S. Zheng, X. Chen, L. Xie, T. Liu, Y. Ren, L. Jin, K. Amine, F. Pan, Y. Xiao, *Nano Energy* **2021**, 88, 106206.

[S7] Y. Shi, Z. Zhang, P. Jiang, A. Gao, K. Li, Q. Zhang, Y. Sun, X. Lu, D. Cao, X. Lu, *Energy Storage Materials* **2021**, 37, 354.

[S8] Y. Cao, Q. Zhang, Y. Wei, Y. Guo, Z. Zhang, W. Huang, K. Yang, W. Chen, T. Zhai, H. Li, Y. Cui, *Advanced Functional Materials* **2020**, 30, 1907023.

[S9] H.-Y. Hu, J.-Y. Li, Y.-F. Liu, Y.-F. Zhu, H.-W. Li, X.-B. Jia, Z.-C. Jian, H.-X. Liu, L.-Y. Kong, Z.-Q. Li, H.-H. Dong, M.-K. Zhang, L. Qiu, J.-Q. Wang, S.-Q. Chen, X.-W. Wu, X.-D. Guo, Y. Xiao, *Chemical Science* **2024**, 15, 5192.

[S10] J. Li, Q. Kuang, N. Wen, H. Yao, J. Wu, Q. Fan, Y. Dong, Y. Zhao, *Journal of Power Sources* **2022**, 521, 230927.
